# Verification of Blood-Brain Barrier Disruption Based on the Clinical Validation Platform Using a Rat Model with Human Skull

**DOI:** 10.3390/brainsci11111429

**Published:** 2021-10-28

**Authors:** Chan Yuk Park, Hyeon Seo, Eun-Hee Lee, Mun Han, Hyojin Choi, Ki-Su Park, Sang-Youl Yoon, Sung Hyun Chang, Juyoung Park

**Affiliations:** 1 Medical Device Development Center, Daegu-Gyeongbuk Medical Innovation Foundation, Daegu 41061, Korea; tanya00@naver.com (C.Y.P.); hseo0612@dgmif.re.kr (H.S.); ehlee@dgmif.re.kr (E.-H.L.); munhan@dgmif.re.kr (M.H.); chn21jin@gmail.com (H.C.); 2 Department of Neurosurgery, School of medicine, Kyungpook National University, Daegu 41944, Korea; kiss798@gmail.com (K.-S.P.); customplus@naver.com (S.-Y.Y.); feeling1398@gmail.com (S.H.C.); 3 Department of High-Tech Medical Device, College of Future Industry, Gachon University, Seongnam-si 13120, Korea

**Keywords:** focused ultrasound, blood-brain barrier, acoustic cavitation, ultrasound field simulation

## Abstract

Methods to improve drug delivery efficiency through blood-brain barrier disruption (BBBD) based on microbubbles and focused ultrasound (FUS) are continuously being studied. However, most studies are being conducted in preclinical trial environments using small animals. The use of the human skull shows differences between the clinical and preclinical trials. BBBD results from preclinical trials are difficult to represent in clinical trials because various distortions of ultrasound by the human skull are excluded in the former. Therefore, in our study, a clinical validation platform based on a preclinical trial environment, using a human skull fragment and a rat model, was developed to induce BBBD under conditions similar to clinical trials. For this, a human skull fragment was inserted between the rat head and a 250 kHz FUS transducer, and optimal ultrasound parameters for the free field (without human skull fragment) and human skull (with human skull fragment) were derived by 300 mV_pp_ and 700 mV_pp_, respectively. BBBD was analyzed according to each case using magnetic resonance images, Evans blue dye, cavitation, and histology. Although it was confirmed using magnetic resonance images and Evans blue dye that a BBB opening was induced in each case, multiple BBB openings were observed in the brain tissues. This phenomenon was analyzed by numerical simulation, and it was confirmed to be due to standing waves owing to the small skull size of the rat model. The stable cavitation doses (SCD_h_ and SCD_u_) in the human skull decreased by 13.6- and 5.3-fold, respectively, compared to those in the free field. Additionally, the inertial cavitation dose in the human skull decreased by 1.05-fold compared to that of the free field. For the histological analysis, although some extravasated red blood cells were observed in each case, it was evaluated as recoverable based on our previous study results. Therefore, our proposed platform can help deduct optimal ultrasound parameters and BBBD results for clinical trials in the preclinical trials with small animals because it considers variables relevant to the human skull.

## 1. Introduction

Various drugs for the treatment of brain diseases such as Alzheimer’s disease, Parkinson’s disease, and brain tumors have been developed; however, the delivery of these drugs into the brain parenchyma through the blood-brain barrier (BBB) is difficult [1,2,3]. Thus, various studies have been conducted to increase drug efficacy by penetrating the BBB for entry into the brain [4,5]. In these studies, BBB disruption (BBBD) with microbubbles and low-intensity focused ultrasound (FUS) has been employed as the safest method, as it is non-invasive and can be repeatedly carried out [6,7].

The effectiveness of BBBD, based on microbubbles and FUS, has been reported in various studies [8,9,10]. Following the injection of microbubbles into blood vessels, FUS is sonicated at the targeted brain blood vessel, and the BBB is temporarily disrupted by the vibration of microbubbles, resulting in improved drug permeability [11,12]. Recently, a few clinical trials have demonstrated that this method is safe and promising for the treatment of brain diseases [13,14].

Currently, most studies on BBBD are being conducted in preclinical trials using small animals such as mice or rats, because the clinical trial application requires extensive validation [15,16,17]. However, it is difficult to directly apply experimental conditions determined by preclinical trials to those for clinical trials, owing to the characteristic nature of the human skull.

According to previous studies, when sonication was performed through a human skull, energy conversion, reflection, and scattering caused ultrasound attenuation. Additionally, it has been reported that strong heating was concentrated at the external edge of the skull, and trabeculae increased according to the reflection and resonance due to internal pressure [18]. Further, changes in the ultrasound focal spot caused by skull thickness differences increased in severity as the center frequency of the transducer increased [19]. Thus, it is difficult to predict the optimal ultrasound power and focal spot distortion for clinical trials from preclinical trial results due to differences in the skull thickness and area between small animals and humans.

It has been reported that in some groups, the ultrasound power level for clinical trials was derived by inserting a human skull over a pig head [20]. However, it is difficult to determine the ultrasound parameters for clinical trials using animal models and human skulls together. Most of the studies related to BBBD have been reported in small animal-based experiments, in vitro experiments, and non-human primate experiments, which do not require a human skull [10,21,22].

In BBBD clinical trials, excessive ultrasound sonication can cause occlusion of the brain blood vessel and lead to a cerebral hemorrhage or stroke. In particular, if excessive ultrasound is sonicated to an area involved in respiration and heartbeat, such as the pons, it can cause severe damage or death by blocking the oxygen supply to the brain [23,24,25].

Therefore, in this study, our goal was to establish a clinical validation platform to obtain optimal ultrasound parameters for clinical trials and predict clinical trial results in the preclinical trial environment. Because a human skull is the most dominant factor causing differences between clinical and preclinical trial results, which affects the ultrasound attenuation ratio and distortion at the focal point and focal depth of the ultrasound beam, the proposed platform uses a human skull and small animal. In particular, a human skull fragment is inserted between an ultrasound transducer and rat head, which helps to determine the proper sonication conditions for clinical trials. Therefore, this approach can minimize the gap between clinical and preclinical trial results.

## 2. Materials and Methods

### 2.1. Study Subjects and the Human Skull

A total of twenty male Sprague–Dawley (SD) rats (8 weeks old and weighing 300 ± 30 g, Koatech, Pyeongtaek, Korea) were used in this study. This study was approved by the Institutional Animal Care and Use Committee of the Daegu-Gyeongbuk Medical Innovation Foundation. A total of four rats were used in the pilot study to define experimental settings for stable BBBD. A total of ten rats were used for the in vivo experiment for BBBD confirmation. A total of two rats were used for cavitation acquisition. The remaining four rats were used for magnetic resonance imaging (MRI) and hematoxylin and eosin (H&E) histology for assessment of any damage in the rat brain. Rats used for our study are listed in Table 1.

The rats were housed in cages at 20−25 °C with a 12-h light to dark cycle. All procedures and handling of the animals were performed according to the ethical guidelines for animal studies.

The human skull fragment, which was 120.54 mm long, 36.53 tall, and 75.55 wide with an average thickness of 5.45 mm, was acquired from an adult male that had undergone craniotomy because of cerebral hemorrhage and edema. The skull was treated with chemicals, and the bones were separated and stored frozen at −20 °C. This study was approved by the institutional review board (IRB) of Kyungpook National University Hospital (IRB no. KNUH 2021-07-028).

### 2.2. Transducer Characteristics

For the BBBD experiment, a focused ultrasound transducer with a spherically curved aperture (center frequency: f_0_, 250 kHz; diameter: 80 mm; focal distance: 100 mm; f-number: 1.2) was used to generate the ultrasound energy. Given that the characteristics of the transducer are one of the main factors affecting BBBD results or cavitation dose analysis, a pulse–echo response test was carried out [26,27]. For this, the transducer was placed in a water bath filled with degassed water, and a reflector made of stainless steel was placed 100 mm away from the transducer surface, considering the focus of the transducer. Using a pulser/receiver system (5072PR, Olympus, Tokyo, Japan), an impulse signal with 80 V_pp_ was applied, and an echo signal was received with a 10-dB receive gain.

### 2.3. BBBD System

**Figure 1:**
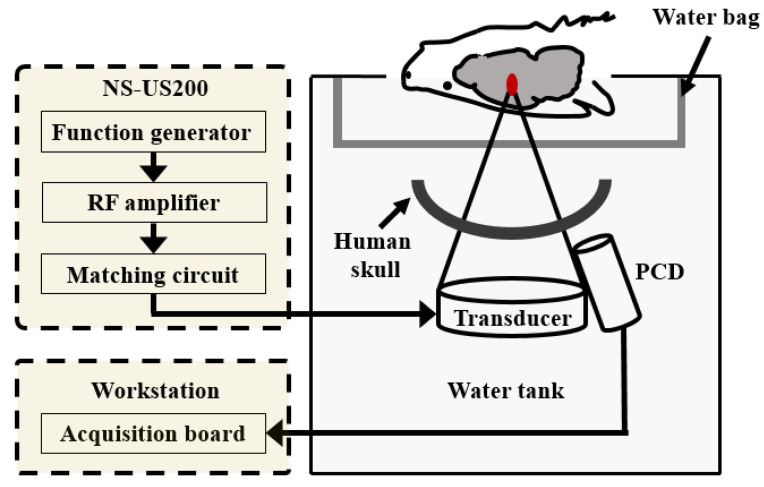
Schematic of the focused ultrasound (FUS) system and experiment environment for blood-brain barrier disruption (BBBD) in the rat. The rat was placed upside down on the surface of the water. The convex part of the human skull was placed toward the transducer similar to that in the clinical trial environment.

An FUS system (NS-US200, NEUROSONA CO., LTD, SEL, South Korea) was used to sonicate the rat brain and to acquire acoustic cavitation emission. Figure 1 shows a schematic of the FUS system and the experimental environment. A 10-ms tone burst 250 kHz sinusoidal wave was generated by the system with a 1-Hz pulse repetition frequency (PRF) for 120 s, and it was amplified through a PRF amplifier and matching circuit. A passive cavitation detector was diagonally placed on the side of the transducer to evaluate the acoustic cavitation emission received from the brain of the rat. Acoustic cavitation signals were recorded on a data acquisition (DAQ) board (ATS460, AlazarTech, Pointe-Claire, QC, Canada; 20 MHz sampling rate, 14 bit, 125 MS/s) in the workstation. The transducer was placed in a water bath filled with degassed water, and the human skull was placed between the transducer and the head of the rat. To prevent irregular reflection and heat damage at the near-field of the transducer, the distance between the inside curvature of the human skull and the head of the rat was set to approximately 60 mm, and the transducer was approximately 40 mm away from the human skull.

### 2.4. Ultrasound Acoustic Characteristics Measurement

**Figure 2:**
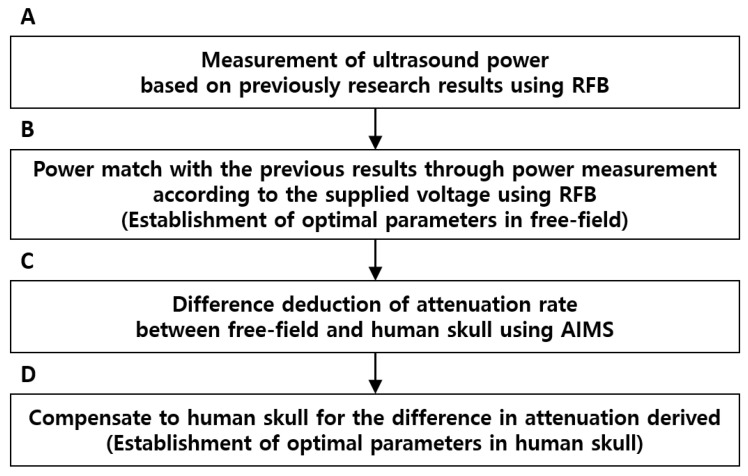
Flow chart for the derivation of optimal ultrasound parameters according to the existence of the human skull. (**A**) Power level derivation of 1 MHz FUS transducer using safe BBBD pressure proven by the previous study. (**B**) Deduction of an optimal supplied voltage of the 250 kHz FUS transducer to match the power of the 1 MHz FUS transducer. (**C**) Measurement of the ultrasound attenuation rate induced by the human skull. (**D**) Free field as much as the attenuation induced by the human skull.

BBBD depends on various factors, such as the microbubble dose and sonication angle [7,28]. The selection of optimal ultrasound parameters is critical for safe BBBD as it determines the acoustic cavitation level by microbubble activity [29]. Excessive cavitation may damage the targeted region; therefore, the optimal ultrasound parameters for the human skull were preferentially analyzed using radiation force balance (RFB, Onda Corporation, Sunnyvale, CA, USA) and an acoustic intensity measurement system (AIMS, Onda Corporation, Sunnyvale, CA, USA). Figure 2 shows the flow chart for the determination of ultrasound parameters in the free field and the human skull. In the free field, a 1 MHz FUS transducer generating 0.6−0.72 MPa at the focus can perform BBBD in the rat brain [30]. Based on previous results, it was verified that driving a 1 kHz tone burst signal with 1 Hz PRF for 10 ms to the ultrasound transducer can generate the same acoustic characteristics as those generated using a 1 MHz transducer. Subsequently, the power measurement of the 250 kHz FUS transducer according to the supplied voltage was performed in the range of 300–900 mV_pp_ to match the ultrasound power of a 1 MHz FUS transducer (Figure 2B), and a signal equal to the RFB measurement of the 1 MHz FUS transducer was supplied. For the human skull, it can be derived by compensating the attenuation rate of ultrasound pressure according to the existence of the skull (Figure 2C). In other words, the attenuation rates of the ultrasound pressure generated in the free field and human skull were measured using AIMS, and ultrasound pressure was compensated in the free field as much as the attenuation induced by the human skull (Figure 2D). Notably, a signal with an amplitude of 300 mV_pp_, frequency of 250 kHz, and one cycle burst with 1 Hz PRF was supplied to the transducer for attenuation measurement.

### 2.5. BBBD Experiment

The procedure for FUS-induced BBBD is shown in Figure 1. The animals were anesthetized with a mixture of Zoletil (25 mg/kg; Virbac Laboratories, Carros, France) and Rumpun (4.6 mg/kg; Bayer, Leverkusen, Germany) and were constantly monitored throughout the experimental procedures. There was no evidence of pain or suffering. The hair on their heads was removed using a shaving razor and hair removal cream. The animals were placed in a supine position on an MR-compatible animal bed.

The BBBD target region was the right caudate putamen (CP). This location was selected for its clinical relevance to neurological disease and to minimize the amount of tissue boundary in the FUS focal area [31]. Before sonication, the microbubbles (0.02 mL/kg, Definity, Lantheus Medical Imaging, North Billerica, MA, USA) were diluted 1:50 in normal saline and injected via a tail vein catheter using an automated syringe pump (Pump 11, Harvard Apparatus, Holliston, MA, USA) for 10 s. This was perfomed to ensure that the circulating microbubbles fully reached the target region. In this case, although it is difficult to directly confirm that the microbubbles had sufficiently reached the target brain region, it can be confirmed indirectly through acoustic cavitation obtained by focused ultrasound sonication.

Thereafter, the parameters selected for safe BBBD in the free field and human skull were applied at the target region (right CP) to induce the FUS–BBBD with the diluted microbubbles over 90 s. The FUS energy was delivered with pulsed sonication consisting of 10-ms tone bursts at a pulse repetition frequency of 1 Hz for 120 s. Following sonication, T1-weighted MR images were obtained with a 0.2 mM/kg gadolinium-based contrast agent (Dotarem, Guerbet, Roissy, France) to confirm BBBD. Evans blue dye (2%; Sigma-Aldrich, St. Louis, MO, USA) was injected intravenously to determine the BBB disruption regions through the brain tissue. All rat brains were perfused and fixed using transcardial perfusion (0.9% normal saline, 200 mL; 4% buffered formalin phosphate, 250 mL). The brains were harvested and processed for H&E staining.

### 2.6. MRI

Imaging was performed using a 3.0 T clinical MRI system (Skyra, Siemens, Erlangen, Germany). T1-weighted contrast-enhanced images were used to evaluate the BBB opening. Susceptibility-weighted imaging (SWI) revealed cerebral microhemorrhage. Edema was evaluated using T2-weighted MR images. The following MRI parameters were employed for two-dimensional turbo spin-echo T1-weighted images: field of view = 40 mm × 40 mm, matrix size = 128 × 128, slice thickness = 1.0 mm, slice gap = 0, repetition time (TR) = 500 ms, echo time (TE) = 6.5 ms, and number of averages = 20. The following parameters were used for T2-weighted images: TR = 2500 ms, TE = 33 ms, number of averages = 20, and the other parameters were equal to those of the T1-weighted images; for SWI: field of view = 50 mm × 50 mm, matrix size = 128 × 128, axial slices = 16, slice thickness = 1.5 mm, slice gap = 0, flip angle = 30, TR = 27 ms, TE = 20 ms, and number of averages = 15. During the MRI scans, the temperature of the animals was maintained at approximately 37 °C using a warm water blanket. ImageJ software (National Institutes of Health, Bethesda, MD, USA) was used for image calculation.

### 2.7. Acoustic Cavitation

To verify the suitability of the selected acoustic parameters considering the existence of the human skull, acoustic cavitation signals emitted from the brain of the rat were observed, because acoustic cavitation signals reveal microbubble activity and tissue damage during the BBBD process. Passive cavitation detection (PCD) (V306, Olympus, Waltham, MA, USA) was used for the acquisition of cavitation signals, and the signals were recorded by using a DAQ board. For the effective acquisition of various frequency components, such as harmonic and ultraharmonics induced from a focused transducer of 250 kHz, a PCD with a center frequency of 1 MHz and a broad bandwidth characteristic (f_1_: 0.47 MHz, f_2_: 3.38 MHz at −20 dB) was used in this study. Cavitation signals were acquired via PCD, transferred to the DAQ board, and recorded in s.

In this study, acoustic cavitation signals were analyzed according to the existence of the human skull, and each case was divided as a base case without microbubble injection to compare cavitation activity by microbubbles. Note that the base case was injected with saline only. In the case of the base, it was conducted for 10 s before microbubble injection. Afterward, microbubble injection was performed for 120 s. Thus, a total of 130 segments (base: 10, microbubble: 120) were recorded for each free field and a human skull.

From these segments, the cavitation dose was derived to quantitatively compare the cavitation activity, and it was classified as the stable cavitation dose (SCD) with harmonic frequencies (*n*f_c_, *n* = 2,3,4, …; SCD_h_) from the transmit frequency (f_c_) SCD with subharmonics (f_c_/2) and ultraharmonics (*n*f_c_/2, *n* = 3,5,7, …; SCD_u_), and inertial cavitation dose (ICD) with broadband noise [32,33]. The calculations were performed using MATLAB (MathWorks Inc., Natick, MA, USA), and the calculation process of the cavitation dose was as described below.

First, base and microbubble segments with time-domain type were converted to frequency spectra using the fast Fourier transform algorithm. To observe the difference of cavitation activity due to microbubbles, all microbubble segments were subtracted from the mean value of the base with the 10 segments. Subsequently, the specific band for SCD_h_ and SCD_u_ were extracted from the frequency spectrum of each segment. In this case, the ICD was defined as all frequency spectrum noises, except the bands of SCD_h_ and SCD_u_. Each cavitation type was calculated using the root-mean-squared area under the frequency spectrum [34,35].

### 2.8. Histology

For H&E staining, SD rats were sacrificed 4 h after BBBD. Harvested brains were embedded in paraffin blocks and serially sectioned at 5 μm thickness in the axial plane. H&E staining was performed on every 50th section (250 μm apart) using an H&E staining kit (Vector Laboratories, Inc., Burlingame, CA, USA). The images were recorded using a slide scanner (Panoramic Scan II, 3D Histech, Budapest, Hungary), and the area of red blood cells (RBCs) in the sonicated brain region was observed. Histological scoring was performed on sonicated regions as well as the contralateral area of the opposite hemisphere. Each section was scored as 0 (*n* = 0, not detected), 1 (0 < *n* ≤ 5), 2 (5 < *n* ≤ 10), or 3 (*n* > 10) according to the number of RBCs.

### 2.9. Statistical Analysis

Statistical analysis was performed using commercial software (IBM Statistical Package for the Social Sciences 21.0, IBM Corp., Armonk, NY, USA). Differences were considered statistically significant at *p* < 0.05.

### 2.10. Numerical Simulations of Acoustic Model

The propagation of FUS was simulated in part of the human skull and in the entire rat head to investigate the transcranial pressure field. Three-dimensional maps of the skull were extracted from a rat micro-computerized tomography (μ CT) (R_mCT2, Rigaku, Japan) scan with $0.08\times 0.08\times 0.08{\;\mathrm{mm}}^{3}$ resolution and a clinical CT scan (Siemens Biograph mCT, Erlange, Germany) for the human skull flap with $0.1\times 0.1\times 0.6\;{\mathrm{mm}}^{3}$ resolution. The images were resampled with linear interpolation at 0.17 mm. The skull layer was segmented using the open-source application of Seg3d, which offers an interactive segmentation function for image data [36]. The simulations were performed using a k-space pseudospectral method-based solver, the k-Wave MATLAB toolbox [37]. A linear simulation was performed based on the assumption that the impact of shear wave propagation was nominal [38,39].

**Figure 3:**
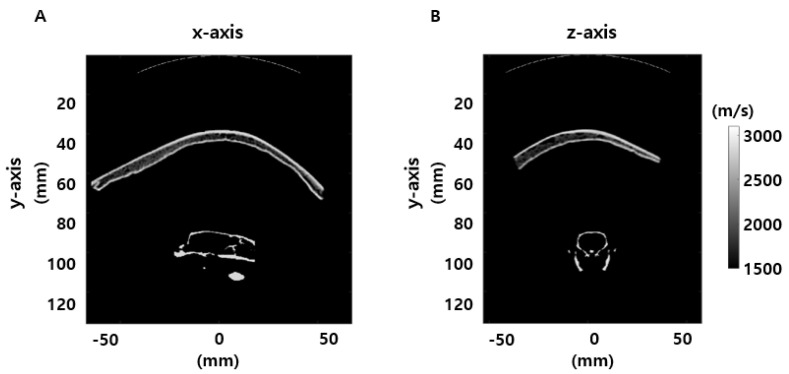
Sound speed field (m/s) for simulations in human skull flap in conjunction with entire rat skull with a representation of the transducer. (**A**) Sagittal view. (**B**) Axial view.

Acoustic simulation with a spherically curved single-element FUS transducer at 250 kHz was modeled in accordance with the transducer configuration used in the experiment. The pulse duration of the sonication was set at 150 μs, and a time step of 16.5 ns was used to compute the simulation. In the simulations, the volume between the skull and the transducer was filled with water. The acoustic properties of the skull and the water are listed in Table 2. For a three-dimensional human skull flap, the heterogeneous acoustic properties of the speed of sound, density, and attenuation were assumed to be proportional to the normalized CT Hounsfield units [40]. For the rat head model, the brain and tissues were assumed to have the sound speed and density of water, and the homogeneous layer of the rat skull was considered [41,42,43]. The simulation setup for the anatomical human skull flap in conjunction with the entire rat skull is plotted for the sound speed field in Figure 3.

## 3. Results

### 3.1. Transducer Characteristics

**Figure 4:**
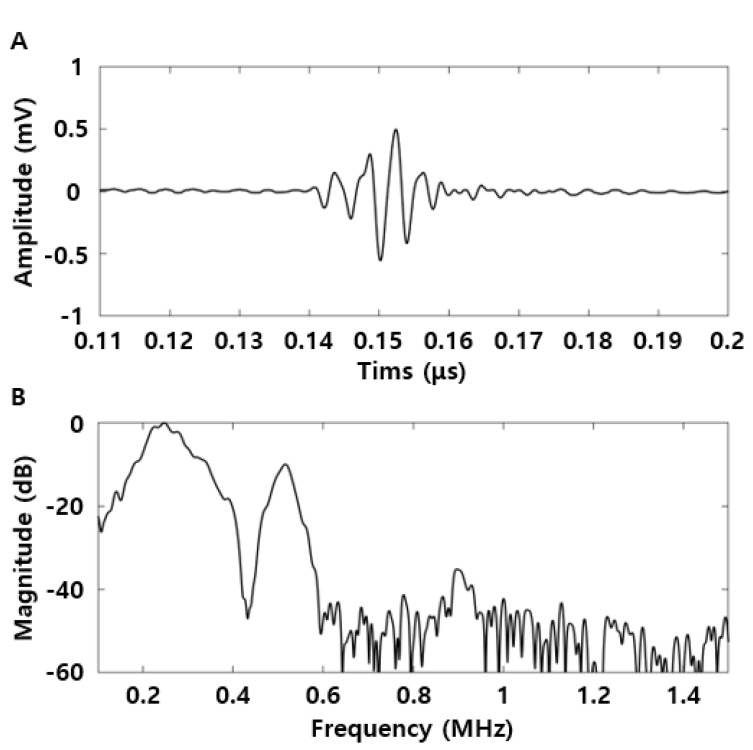
Pulse-echo response of focused ultrasound transducer. (**A**) Time-domain and (**B**) frequency spectrum results.

Figure 4 shows the time-domain waveform and its frequency-domain spectrum. The amplitude of the measured echo signal was 1.0 Vpp at approximately 0.14 ms (Figure 4A). In the frequency spectrum, the fundamental frequency (f_0_; 250 kHz) and its second harmonic frequency (2f_0_; 500 kHz) components were sequentially high, and the magnitude difference between the two frequencies was approximately 10 dB (Figure 4B).

### 3.2. Ultrasound Acoustic Characteristic Analysis According to Existence of Human Skull

**Figure 5:**
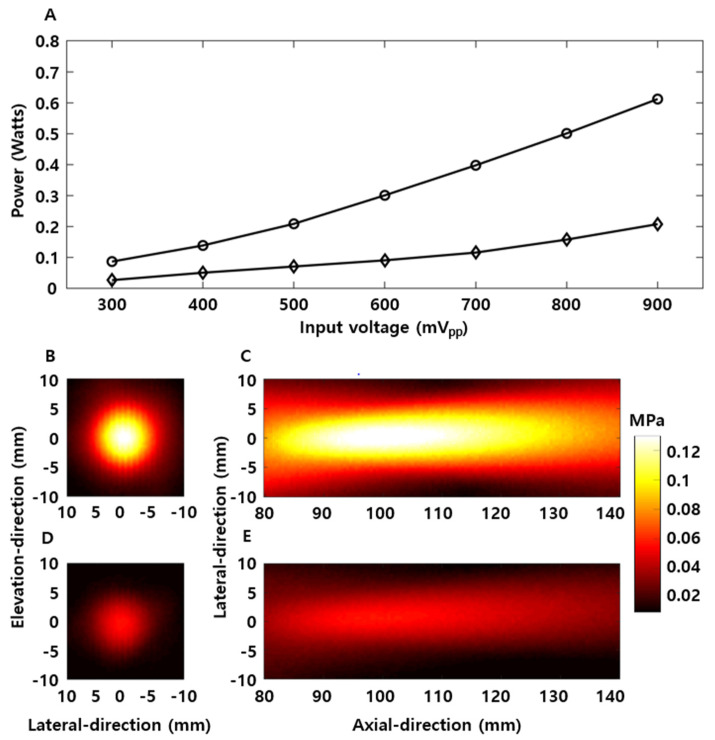
Measurement results of the FUS transducer for deduction of optimal input voltage. (**A**) Relationship between voltage and power of the 250 kHz FUS transducer (circle: free field, diamond: human skull). (**B**,**C**) Acoustic beam profile in the free field. (**D**,**E**) Acoustic beam profile in the human skull.

Ultrasound parameters for safe BBBD in the free field and human skull were determined based on our previous study using a 1 MHz FUS transducer. First, an ultrasound power of 0.7 MPa using a 1 MHz FUS transducer was measured as 0.09 W. When 300 mV_pp_ input voltage was supplied to a 250 kHz FUS transducer, its ultrasound power was measured as 0.087 W, which is close to 0.09 W. Thus, 300 mV_pp_ was selected as an ultrasound parameter for safe BBBD in the free field (Figure 5A).

The attenuation rate was measured according to the existence of the human skull, using equal input parameters (300 mV_pp_), and was compensated for based on the attenuation rate in the human skull. For this, a hydrophone was placed inside the human skull, and a 1 MHz FUS transducer was located outside of the skull. The maximum intensities of the free field and the human skull were measured as 0.26 MPa and 0.12 MPa, respectively (Figure 5B–E). Thus, it was confirmed that an attenuation rate of approximately 54% was observed for the human skull, and 700 mV_pp_ was selected as the optimal input voltage for the human skull to compensate for attenuation. It was confirmed that a driving voltage of 700 mV_pp_ resulted in 0.116 W of ultrasonic power when considering the human skull.

### 3.3. BBBD

**Figure 6:**
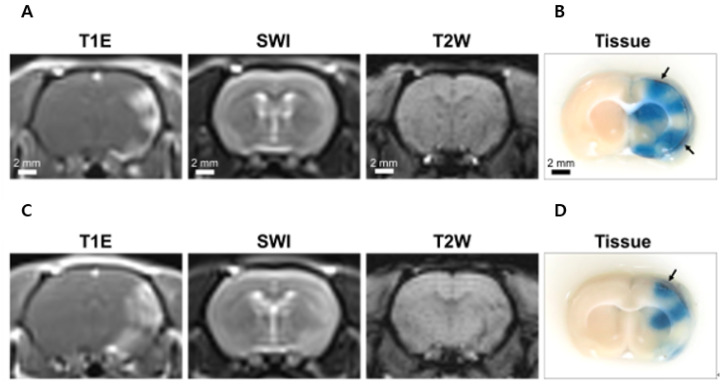
Representative magnetic resonance (MR) images and Evans blue dye-stained brain sections after FUS-induced the BBB opening in rats. (**A**) BBB opening MR result without the human skull for 300 mV input voltage. T1E MR confirmed the BBB opening. T2W and susceptibility-weighted imaging (SWI) MR images were used to evaluate edema and cerebral microhemorrhages, respectively. (**B**) Evans blue dye-stained tissue image confirmed the BBB opening and extravasated red blood cells (RBCs). (**C**) BBB opening MR images and (**D**) Evans blue dye-stained tissue image with the human skull for 700 mV input voltage. Note that areas where a small amount of RBCs were visible are indicated by black arrows.

In this study, we induced a BBB opening with two FUS parameters (free field, without human skull, 300 mV_pp_; human skull, 700 mV_pp_). The FUS-induced BBB opening at targeted brain regions was confirmed using T1-weighted contrast-enhanced images and Evans blue dye-stained brain section images (Figure 6). The MR signal intensity under sonication conditions was higher than that in the contralateral region in the T1E images. T2W and SWI MR images were used to evaluate the edema and cerebral microhemorrhages (Figure 6A,C), respectively. Microscopic edema and cerebral microhemorrhages were observed in both images. In addition, it was confirmed that the BBB opening was in the Evans blue dye-stained brain section image (Figure 6B,D). Interestingly, Figure 6B,D show Evans blue dye leakage at multiple foci. We carried out numerical simulations to explain this phenomenon. The results of the simulations are presented in detail in Section 3.6, Acoustic Simulation.

### 3.4. Passive Acoustic Cavitation

**Figure 7:**
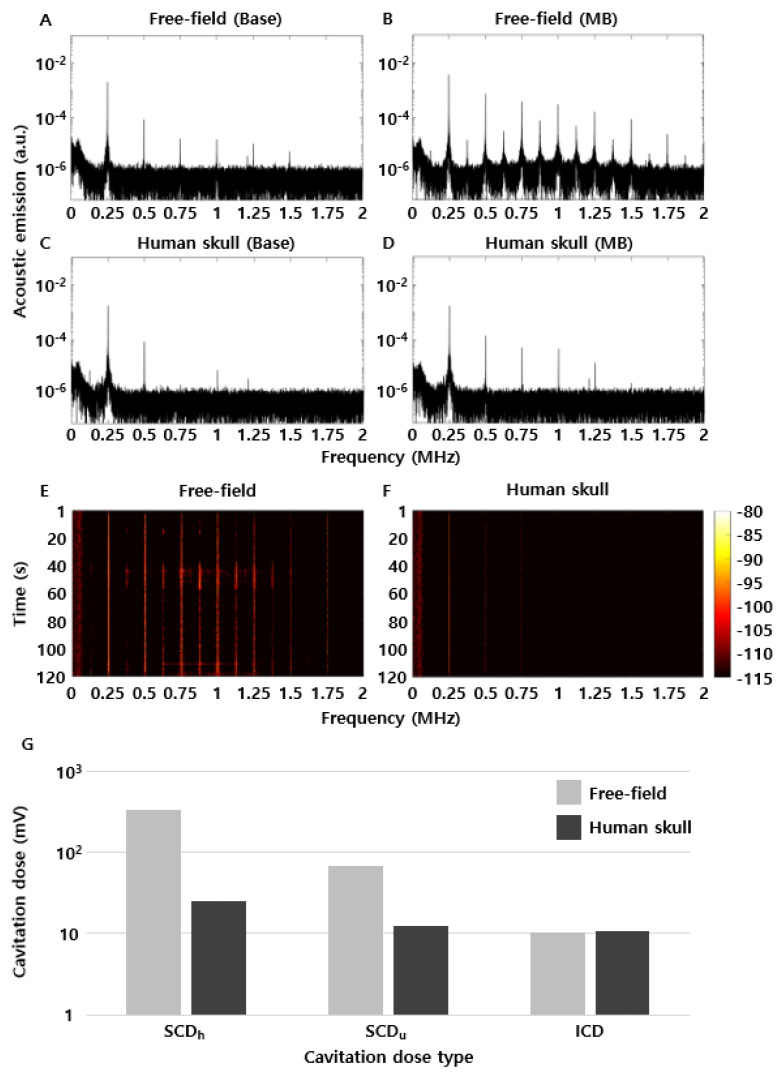
Acoustic cavitation results according to analysis method. (**A**–**D**) Representative results of acoustic cavitation. (**E**,**F**) Spectrogram results of acoustic cavitation acquired during the total experimental time. (**G**) Cavitation dose results by cavitation type.

Acoustic cavitation was analyzed using frequency spectra, spectrograms, and cavitation doses, as shown in Figure 7. Figure 7A–D show the representative frequency spectrum results in the free field (Figure 7A,B) and human skull (Figure 7C,D). In Figure 7A, in the free field without microbubbles, harmonic components were received because of the nonlinear phenomenon only in the medium. However, when microbubbles were injected (Figure 7B), harmonic components were obtained with a much higher level than that of the free field without microbubbles case because of the cavitation effect of the microbubbles. Additionally, the occurrence of ultraharmonic components and broadband noise over the approximate 0.5–1.5 MHz spectrum indicates that vigorous cavitation activity was generated. Subsequently, spectrograms were observed in the human skull case (Figure 7C,D). As expected, the harmonic components declined because of the reflection and attenuation of the ultrasonic beam by the human skull; however, the number of harmonic components and acoustic emissions were high in the presence of microbubbles, compared to that in the base case.

Figure 7A–D are representative images of acoustic cavitation. In other words, they are results captured in the middle of microbubble injection for 120 s. During the injection, the magnitude of harmonic components was continuously changed every second. Thus, analysis of the frequency characteristics is important during the 120 s using spectrogram. In the case of the free field in the spectrogram, harmonic characteristics similar to Figure 7B were generated for approximately 40–60 s. However, sub-harmonic characteristics were not observed at other times. This means that the microbubbles were most active for approximately 40–60 s in the brains of rats. Therefore, it is important to analyze the cavitation using spectrogram and cavitation dose parameters.

These frequency spectra are shown in more detail in the spectrogram derived from the microbubbles of the free field (Figure 7E) and human skull (Figure 7F). Harmonic components were observed in the free field during the experiment, and ultraharmonic components were observed in a bandwidth of approximately 0.625–1.375 MHz. However, it was difficult to observe harmonic components in the human skull at the same magnitude scale as that of the free field.

These cavitation characteristics were quantified using the cavitation dose (Figure 7G). The cavitation dose emissions of SCD_h_, SCD_u_, and ICD in the free field were 340.0 mV, 67.6 mV, and 10.4 mV, and those in the human skull were 25.1 mV, 12.7 mV, 10.9 mV, respectively. Thus, SCD_h_ and SCD_u_ in the human skull decreased by 13.6- and 5.3-fold, respectively, compared to those in the free field, and ICD in the human skull increased by 1.05-fold compared to that in the free field. These results are similar to those of a previous BBBD ex vivo study that utilized a human skull fragment.

### 3.5. Histological Analysis

**Figure 8:**
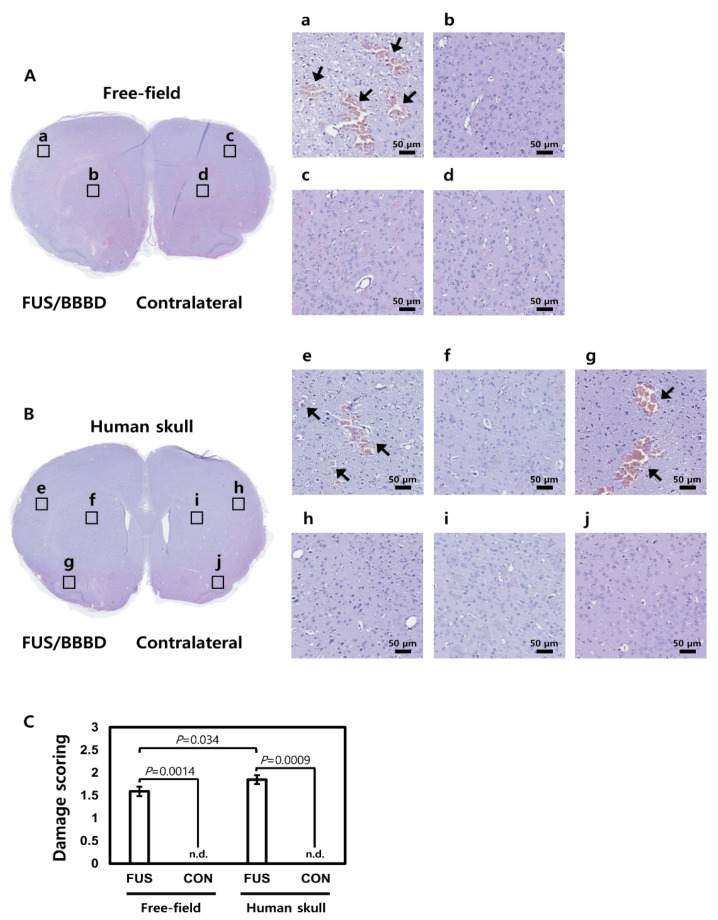
Histological analysis of the rat brain sections after FUS induced the BBB opening using hematoxylin and eosin (H&E) staining. (**A**) The upper brain was sonicated without the human skull. The enlarged images showed (**a**,**b**) FUS/BBBD regions and (**c**,**d**) contralateral regions (scale bar = 50 μm). (**B**) The bottom brain was sonicated with the human skull. The enlarged images showed FUS/BBBD regions (**e**–**g**) and contralateral regions (**h**–**j**) (scale bar = 50 μm). The black arrow shows cerebral microhemorrhages induced by FUS. (**C**) The damage score of H&E staining for each of the FUS groups was represented by a bar graph. All data are presented as the mean ± standard deviation (*n* = 3 slides). n.d., not detected. Analysis of variance with a multiple comparisons test was performed, and the results are as follows: comparison with contralateral versus FUS/BBBD (*p* = 0.0014 in the free field, *p* = 0.0009 in the human skull) and FUS/BBBD in the free field versus FUS/BBBD in the human skull (*p* = 0.034).

H&E histological analysis of the acute specimens revealed that sonication resulted in either no apparent change in the tissue or some extravasated RBCs, as shown in Figure 8. Only a few spots had regions with a small number of RBCs, indicated by black arrows in Figure 8, without visible large vessel ruptures. We performed a histological assessment to compare tissue damage according to distinct FUS parameters. The characteristics of lesions induced by BBBD were similar in both tissue slices. As shown in Figure 8, a few RBC extravasations were detected in the cortex of the sonicated region (Figure 8a,e,g). Within a lesion, the microvacuolation of tissue was not observed in either condition (Figure 8a–j). In the parenchyma region, the tissues had no significant extravasated RBCs or microvacuolation (Figure 8b,g). The histological results supported the similarity of the applied ultrasound parameters with and without human skulls.

### 3.6. Acoustic Simulation

**Figure 9:**
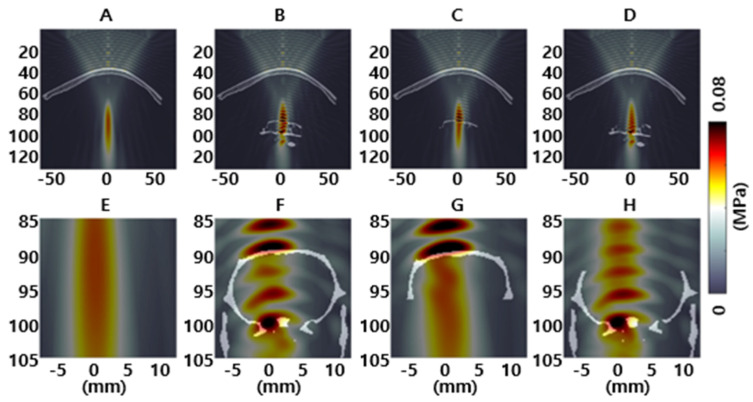
Acoustic distributions at 250 kHz are depicted in (**A**–**D**) sagittal and (**E**–**H**) coronal views after transcranial transmission of the (**A**,**E**) human skull (**B**,**F**) human with rat skull, and human skull fragment with rat skull excluding their (**C**,**G**) bottom or (**D**,**H**) upper halves.

Numerical simulations of the acoustic field were performed to estimate the spatial profiles of peak pressure after transmission through the human skull with and without the rat skull, as shown in Figure 9. In the case of the human skull with a full rat skull, by reproducing the optimal clinical imitation platform, we found considerable interference patterns with increased peak pressure (0.084 MPa) inside the rat brain when compared with the transmission through the human skull without the rat skull (maximum pressure: 0.060 MPa). Owing to the importance of rat skull geometry on acoustic interference, numerical simulations were performed on the human skull with the rat skull, excluding their bottom or upper halves (Figure 9C,D,G,H). In the human skull with a baseless rat skull model, we found a comparable intracranial pressure field with the human skull model, resulting in an intracranial maximum pressure of 0.061 MPa. When we excluded the upper part of the rat skull, we found interference patterns that were comparable to the transcranial transmission of both human and entire rat skulls. These results suggest that rat skull base reflections have a major influence on the interference patterns of the intracranial pressure field.

## 4. Discussion

The BBB is an impermeable physical barrier in cerebral blood vessels, consisting of vascular endothelial cells supported by tight junctions, a basement membrane, pericytes, and neural cells. The BBB is essential in protecting the brain from toxins and controlling cerebral homeostasis. However, it is also considered a major obstacle for drug delivery in the treatment of neurological disorders and brain tumors [48,49]. FUS combined with microbubbles is considered a promising method to treat these diseases because it can improve the efficacy of drug delivery to the brain non-invasively and target-specifically. Although this technique has been applied to a few clinical trials thus far, most of the research has been conducted as preclinical trials and using small animals. However, a primary difference in clinical and preclinical environments is the existence of a human skull. The human skull is thicker than that of a rat, and its thickness is not uniform, which results in insufficient ultrasound energy at the focal point, a distorted focal region, and a change in the focal depth due to acoustic attenuation and refraction at the skull. Therefore, there is a large gap in the optimal sonication parameters between clinical and preclinical trials, which leads to inconsistencies between the results of clinical and preclinical trials. To minimize this gap, we constructed a platform that can offer optimal ultrasound parameters for clinical trials by using a human skull. The proposed platform can be used to predict the results of clinical trials in a preclinical environment. In our previous study, the deduction methods of optimal ultrasound parameters were shown for free field and human skull cases, based on results using a 1 MHz FUS transducer. Given that each transducer has a different resonance frequency, it is not possible to derive a parameter for safety, which is the power per unit area. It must be matched to power, which is the energy per unit of time [50]. These methods are expected to be utilized as a method for optimizing the parameter derivation between different ultrasound transducers. The optimal input voltages in the free field and human skull using a 250 kHz FUS transducer were selected as 300 mV_pp_ (0.087 W) and 700 mV_pp_ (0.116 W), respectively, via the platform, and a 54% attenuation rate was shown, which is consistent with other studies on BBBD through the human skull. These attenuation rates are similar to those of other experimental results using the human skull [51].

Among the experimental results, SCD_h_ and SCD_u_ in the human skull decreased by 13.6- and 5.3-fold compared to those in the free field; however, ICD in the human skull increased by 1.05-fold compared to that in the free field. Generally, the SCD series and ICD are used as indicators to monitor the conditions of microbubbles. Specifically, SCD is used to monitor the stability of microbubbles, and ICD is used to observe the inertial conditions of microbubbles [22,52]. Considering this, SCD_h_ and SCD_u_ showed a large difference according to the existence of the human skull, whereas ICD in the human skull showed a similar value to that in the free field. This means that the ICD dose can be used to predict the inertial cavitation characteristics in clinical trials. In other words, the free field and human skull were assumed to be preclinical and clinical trial environments, respectively, in our experiment. If the ICD doses are similar in both cases, the derived guidelines from preclinical trials can be directly applied in clinical trials. Although our acoustic cavitation dose results may not have statistical significance, the trend according to the existence of the human skull is consistent with prior results.

The second harmonic frequency of the ultrasound transducer used in this study had a magnitude of approximately −10 dB. A quantitative analysis of the cavitation dose according to the magnitude of this harmonic frequency is necessary for accurate BBBD evaluation.

When analyzing the MR images, Evans blue dye, and histology based on the cavitation dose, BBBD was observed. Indeed, we studied the long-term effects of BBBD in our previous study, showing that the brain was restored to a normal state within 72 h after BBBD [53]. Furthermore, our ultrasound parameters were derived based on previous results using a 1 MHz FUS transducer. Based on these parameters, if precise output control of the transducer is performed, safe BBBD induction is possible.

Interference patterns were observed in MR images and Evans blue dye. To analyze the cause of this phenomenon, four numerical simulations were performed: one behind the human skull, one behind the human skull with the rat skull (reproducing the optimal experiments), one behind the human skull with the upper half of the rat skull, and one behind the human skull with the bottom half of the rat skull. Using different simulation settings, we explored the impact of the existence of the rat skull on the clinical validation platform. The spatial profiles of the peak pressure are shown in Figure 9A,B for the human skull with and without a full rat skull. The human skull with a full rat skull showed considerable interference patterns with increased intracranial peak pressure. These patterns arose in the full rat skull and the bottom part as well, suggesting that the bottom part of the rat skull was the primary cause of standing waves. Furthermore, earlier simulation studies on low-frequency FUS have shown that the acoustic field is much more complex than a single localized spot at the geometric focus because of the presence of standing waves [54,55]. Constans et al. investigated the effects of low-intensity FUS after transmission through rat and macaques using a quadri-band transducer capable of operating at 200 kHz, 320 kHz, 850 kHz, and 1380 kHz and reported a 4-fold higher ratio of the standing wave at 200 kHz compared to 1380 kHz in the rat. Similarly, Younan et al. reported a 1.8-fold increase in peak pressure inside the rat head compared to the free field, owing to the reverberations of the ultrasound wave [54]. Therefore, interference patterns with increased pressure in the human and rat skull models were owing to the small thickness of the rat skull with respect to wavelength; thus, the interference patterns should be much lower in the full human skull because of its larger size. For this reason, although minor edema and microhemorrhage in the brain were observed in our study, it is expected that the risk to humans is negligible.

In our experiment, a limited number of subjects was used for cavitation acquisition, MRI, and histology. Therefore, although our experimental results agree with those of earlier studies, further studies are required to secure statistically significant results.

## 5. Conclusions

BBBD was analyzed according to the existence of the human skull using the developed clinical validation platform. In our study, the difference in the human skull was confirmed using MR images and cavitation dose. Some damage occurred in the brain tissue; however, we also proposed a method to optimize ultrasound parameters that can minimize this damage. Although the interference pattern was generated by the rat skull because of its small size and low center frequency, if the experiment is conducted using medium or large animals, it is expected that more accurate BBBD results can be obtained using our platform because of the similarity in size of the target and human skulls.

## Figures and Tables

**Table 1:** Summary of Animal Numbers.

Experiments	Pilot Study	Evans Blue for BBBD Confirmation	Cavitation Acquisition	MRI and Histology
Animal number	4	10	2	4

**Table 2:** Acoustic parameters used for simulation design [44,45,46,47].

Speed of Sound (m s^−1^)	Density (kg m^−3^)	Attenuation (dB MHz−1.43 cm^−1^)
$c_{water}=1482$	$\rho_{water}=1000$	$\alpha_{water}=0.24\times 10^{-2}$
$c_{skull,\;homo}=2850$	$\rho_{skull,homo}=1732$	$\alpha_{skull,homo}=8.83$
$c_{skull,hetero}=3100$	$\rho_{skull,hetero}=2200$	$\alpha_{min,skull,hetero}=12.67$ $,\;\alpha_{max,skull,hetero}=51.42$

## References

[1] Upadhyay R.K. (2014). Drug Delivery Systems, CNS Protection, and the Blood Brain Barrier. BioMed Res. Int..

[2] Dong X. (2018). Current Strategies for Brain Drug Delivery. Theranostics.

[3] Naqvi S., Panghal A., Flora S.J.S. (2020). Nanotechnology: A Promising Approach for Delivery of Neuroprotective Drugs. Front. Neurosci..

[4] Salcman M., Samaras G.M. (1983). Interstitial microwave hyperthermia for brain tumors. J. Neuro-Oncol..

[5] Kumagai A.K., Eisenberg J.B., Pardridge W. (1987). Absorptive-mediated endocytosis of cationized albumin and a beta-endorphin-cationized albumin chimeric peptide by isolated brain capillaries. Model system of blood-brain barrier transport. J. Biol. Chem..

[6] Hynynen K., McDannold N., Vykhodtseva N., Jolesz F.A. (2001). Noninvasive MR Imaging–guided Focal Opening of the Blood-Brain Barrier in Rabbits. Radiology.

[7] Song K.-H., Fan A.C., Hinkle J.J., Newman J., Borden M.A., Harvey B.K. (2017). Microbubble gas volume: A unifying dose parameter in blood-brain barrier opening by focused ultrasound. Theranostics.

[8] Aryal M., Fischer K., Gentile C., Gitto S., Zhang Y.-Z., McDannold N. (2017). Effects on P-Glycoprotein Expression after Blood-Brain Barrier Disruption Using Focused Ultrasound and Microbubbles. PLoS ONE.

[9] Song K.-H., Harvey B.K., Borden M.A. (2018). State-of-the-art of microbubble-assisted blood-brain barrier disruption. Theranostics.

[10] Chen K.-T., Wei K.-C., Liu H.-L. (2019). Theranostic Strategy of Focused Ultrasound Induced Blood-Brain Barrier Opening for CNS Disease Treatment. Front. Pharmacol..

[11] Fan C.-H., Lin C.-Y., Liu H.-L., Yeh C.-K. (2017). Ultrasound targeted CNS gene delivery for Parkinson’s disease treatment. J. Control. Release.

[12] Park B., Yoon S., Choi Y., Jang J., Park S., Choi J. (2020). Stability of Engineered Micro or Nanobubbles for Biomedical Applications. Pharmaceutics.

[13] Gasca-Salas C., Fernández-Rodríguez B., Pineda-Pardo J.A., Rodríguez-Rojas R., Obeso I., Hernández-Fernández F., del Álamo M., Mata D., Guida P., Ordás-Bandera C. (2021). Blood-brain barrier opening with focused ultrasound in Parkinson’s disease dementia. Nat. Commun..

[14] Mehta R.I., Carpenter J.S., Mehta R.I., Haut M.W., Ranjan M., Najib U., Lockman P., Wang P., D’Haese P.-F., Rezai A.R. (2021). Blood-Brain Barrier Opening with MRI-guided Focused Ultrasound Elicits Meningeal Venous Permeability in Humans with Early Alzheimer Disease. Radiology.

[15] Timbie K.F., Mead B.P., Price R.J. (2015). Drug and gene delivery across the blood–brain barrier with focused ultrasound. J. Control. Release.

[16] Cho H., Lee H.-Y., Han M., Choi J.-R., Ahn S., Lee T., Chang Y., Park J. (2016). Localized Down-regulation of P-glycoprotein by Focused Ultrasound and Microbubbles induced Blood-Brain Barrier Disruption in Rat Brain. Sci. Rep..

[17] McMahon D., Bendayan R., Hynynen K. (2017). Acute effects of focused ultrasound-induced increases in blood-brain barrier permeability on rat microvascular transcriptome. Sci. Rep..

[18] Pinton G., Aubry J.-F., Bossy E., Muller M., Pernot M., Tanter M. (2011). Attenuation, scattering, and absorption of ultrasound in the skull bone. Med. Phys..

[19] Hynynen K., A Jolesz F. (1998). Demonstration of Potential Noninvasive Ultrasound Brain Therapy Through an Intact Skull. Ultrasound Med. Biol..

[20] Huang Y., Alkins R., Schwartz M.L., Hynynen K. (2017). Opening the Blood-Brain Barrier with MR Imaging–guided Focused Ultrasound: Preclinical Testing on a Trans–Human Skull Porcine Model. Radiology.

[21] Conti A., Kamimura H.A.S., Novell A., Duggento A., Toschi N. (2020). Magnetic Resonance Methods for Focused Ultrasound-Induced Blood-Brain Barrier Opening. Front. Phys..

[22] Pouliopoulos A.N., Wu S.-Y., Burgess M.T., Karakatsani M.E., Kamimura H., Konofagou E.E. (2019). A Clinical System for Non-invasive Blood–Brain Barrier Opening Using a Neuronavigation-Guided Single-Element Focused Ultrasound Transducer. Ultrasound Med. Biol..

[23] Neuwelt E.A., Glasberg M., Frenkel E., Barnett P. (1983). Neurotoxicity of chemotherapeutic agents after blood-brain barrier modification: Neuropathological studies. Ann. Neurol..

[24] Kondo A., Inoue T., Nagara H., Tateishi J., Fukui M. (1987). Neurotoxicity of adriamycin passed through the transiently disrupted blood-brain barrier by mannitol in the rat brain. Brain Res..

[25] Carpentier A., Canney M., Vignot A., Reina V., Beccaria K., Horodyckid C., Karachi C., Leclercq D., Lafon C., Chapelon J.-Y. (2016). Clinical trial of blood-brain barrier disruption by pulsed ultrasound. Sci. Transl. Med..

[26] Fei C., Ma J., Chiu C.T., Williams J.A., Fong W., Chen Z., Zhu B., Xiong R., Shi J., Hsiai T.K. (2015). Design of matching layers for high-frequency ultrasonic transducers. Appl. Phys. Lett..

[27] Sung J.H., Jeong J.S. (2018). Development of High-Frequency (>60 MHz) Intravascular Ultrasound (IVUS) Transducer by Using Asymmetric Electrodes for Improved Beam Profile. Sensors.

[28] Huh H., Park T.Y., Seo H., Han M., Jung B., Choi H.J., Lee E.-H., Pahk K.J., Kim H., Park J. (2020). A local difference in blood–brain barrier permeability in the caudate putamen and thalamus of a rat brain induced by focused ultrasound. Sci. Rep..

[29] Burgess A., Shah K., Hough O., Hynynen K. (2015). Focused ultrasound-mediated drug delivery through the blood–brain barrier. Expert Rev. Neurother..

[30] Jung B., Huh H., Lee E.-H., Han M., Park J. (2019). An advanced focused ultrasound protocol improves the blood-brain barrier permeability and doxorubicin delivery into the rat brain. J. Control. Release.

[31] Valdez M.A., Fernandez E., Matsunaga T., Erickson R.P., Trouard T.P. (2020). Distribution and Diffusion of Macromolecule Delivery to the Brain via Focused Ultrasound using Magnetic Resonance and Multispectral Fluorescence Imaging. Ultrasound Med. Biol..

[32] Chu P.-C., Chai W.-Y., Tsai C.-H., Kang S.-T., Yeh C.-K., Liu H.-L. (2016). Focused Ultrasound-Induced Blood-Brain Barrier Opening: Association with Mechanical Index and Cavitation Index Analyzed by Dynamic Contrast-Enhanced Magnetic-Resonance Imaging. Sci. Rep..

[33] Yang Y., Zhang X., Ye D., Laforest R., Williamson J., Liu Y., Chen H. (2019). Cavitation dose painting for focused ultrasound-induced blood-brain barrier disruption. Sci. Rep..

[34] Qiu Y., Luo Y., Zhang Y., Cui W., Zhang D., Wu J., Zhang J., Tu J. (2010). The correlation between acoustic cavitation and sonoporation involved in ultrasound-mediated DNA transfection with polyethylenimine (PEI) in vitro. J. Control. Release.

[35] Xu S., Zong Y., Feng Y., Liu R., Liu X., Hu Y., Han S., Wan M. (2014). Dependence of pulsed focused ultrasound induced thrombolysis on duty cycle and cavitation bubble size distribution. Ultrason. Sonochem..

[36] Scientific Computing and Imaging Institute (SCI Institute) “Seg3D” Volumetric Image Segmentation and Visualization. SCAI Institute. www.seg3d.org.

[37] Cox B.T., Kara S., Arridge S., Beard P.C. (2007). k-space propagation models for acoustically heterogeneous media: Application to biomedical photoacoustics. J. Acoust. Soc. Am..

[38] Fry F.J., Barger J.E. (1978). Acoustical properties of the human skull. J. Acoust. Soc. Am..

[39] Narumi R., Matsuki K., Mitarai S., Azuma T., Okita K., Sasaki A., Yoshinaka K., Takagi S., Matsumoto Y. (2013). Focus Control Aided by Numerical Simulation in Heterogeneous Media for High-Intensity Focused Ultrasound Treatment. Jpn. J. Appl. Phys..

[40] Aubry J.-F., Tanter M., Pernot M., Thomas J.-L., Fink M. (2003). Experimental demonstration of noninvasive transskull adaptive focusing based on prior computed tomography scans. J. Acoust. Soc. Am..

[41] Mueller J.K., Ai L., Bansal P., Legon W. (2016). Computational exploration of wave propagation and heating from transcranial focused ultrasound for neuromodulation. J. Neural Eng..

[42] Mueller J.K., Ai L., Bansal P., Legon W. (2017). Numerical evaluation of the skull for human neuromodulation with transcranial focused ultrasound. J. Neural Eng..

[43] Constans C., Mateo P., Tanter M., Aubry J.-F. (2017). Potential impact of thermal effects during ultrasonic neurostimulation: Retrospective numerical estimation of temperature elevation in seven rodent setups. Phys. Med. Biol..

[44] Connor C.W. (2005). Simulation Methods and Tissue Property Models for Non-Invasive Transcranial Focused Ultrasound Surgery.

[45] White P.J., Clement G.T., Hynynen K. (2006). Longitudinal and shear mode ultrasound propagation in human skull bone. Ultrasound Med. Biol..

[46] Marquet F., Pernot M., Aubry J.-F., Montaldo G., Marsac L., Tanter M., Fink M. (2009). Non-invasive transcranial ultrasound therapy based on a 3D CT scan: Protocol validation andin vitroresults. Phys. Med. Biol..

[47] Treeby B.E., Cox B.T. (2014). Modeling power law absorption and dispersion in viscoelastic solids using a split-field and the fractional Laplacian. J. Acoust. Soc. Am..

[48] Galstyan A., Markman J.L., Shatalova E.S., Chiechi A., Korman A.J., Patil R., Klymyshyn D., Tourtellotte W.G., Israel L.L., Braubach O. (2019). Blood–brain barrier permeable nano immunoconjugates induce local immune responses for glioma therapy. Nat. Commun..

[49] Montemurro N., Perrini P., Rapone B. (2020). Clinical risk and overall survival in patients with diabetes mellitus, hyperglycemia and glioblastoma multiforme. A review of the current literature. Int. J. Environ. Res. Public Health.

[50] Mamvura T.A., Iyuke S.E., Paterson A.E. (2018). Energy changes during use of high-power ultrasound on food grade surfaces. S. Afr. J. Chem. Eng..

[51] Phipps M.A., Jonathan S.V., Yang P.-F., Chaplin V., Chen L.M., Grissom W.A., Caskey C.F. (2019). Considerations for ultrasound exposure during transcranial MR acoustic radiation force imaging. Sci. Rep..

[52] O’Reilly M.A., Hynynen K. (2012). Blood-Brain Barrier: Real-time Feedback-controlled Focused Ultrasound Disruption by Using an Acoustic Emissions–based Controller. Radiology.

[53] Han M., Seo H., Choi H., Lee E.-H., Park J. (2021). Localized Modification of Water Molecule Transport after Focused Ultrasound-Induced Blood–Brain Barrier Disruption in Rat Brain. Front. Neurosci..

[54] Younan Y., Deffieux T., Larrat B., Fink M., Tanter M., Aubry J.-F. (2013). Influence of the pressure field distribution in transcranial ultrasonic neurostimulation. Med. Phys..

[55] Constans C., Deffieux T., Pouget P., Tanter M., Aubry J.-F. (2017). A 200–1380-kHz Quadrifrequency Focused Ultrasound Transducer for Neurostimulation in Rodents and Primates: Transcranial In Vitro Calibration and Numerical Study of the Influence of Skull Cavity. IEEE Trans. Ultrason. Ferroelectr. Freq. Control..

